# The molecular mechanism of the type IVa pilus motors

**DOI:** 10.1038/ncomms15091

**Published:** 2017-05-05

**Authors:** Matthew McCallum, Stephanie Tammam, Ahmad Khan, Lori L. Burrows, P. Lynne Howell

**Affiliations:** 1 Department of Biochemistry, University of Toronto, Toronto, Ontario, Canada M5S 1A8; 2 Program in Molecular Medicine, Peter Gilgan Centre for Research and Learning, The Hospital for Sick Children, Toronto, Ontario, Canada M5G 0A4; 3 Department of Biochemistry and Biomedical Sciences and the Michael G. DeGroote Institute for Infectious Disease Research, McMaster University, Hamilton, Ontario, Canada L8N 3Z5

## Abstract

Type IVa pili are protein filaments essential for virulence in many bacterial pathogens; they extend and retract from the surface of bacterial cells to pull the bacteria forward. The motor ATPase PilB powers pilus assembly. Here we report the structures of the core ATPase domains of *Geobacter metallireducens* PilB bound to ADP and the non-hydrolysable ATP analogue, AMP-PNP, at 3.4 and 2.3 Å resolution, respectively. These structures reveal important differences in nucleotide binding between chains. Analysis of these differences reveals the sequential turnover of nucleotide, and the corresponding domain movements. Our data suggest a clockwise rotation of the central sub-pores of PilB, which through interactions with PilC, would support the assembly of a right-handed helical pilus. Our analysis also suggests a counterclockwise rotation of the C2 symmetric PilT that would enable right-handed pilus disassembly. The proposed model provides insight into how this family of ATPases can power pilus extension and retraction.

## Abstract (web summary)

Bacterial type IVa pili are protein filaments used for motility and protein secretion. Here the authors present crystal structures of the *Geobacter metallireducens* PilB ATPase in two nucleotide states, and suggest a clockwise rotation of the central sub-pores of PilB that would support the assembly of a right-handed helical pilus.

The Type IVa Pilus (T4aP) is a protein grappling hook that can pull bacteria forward with forces in excess of 100 pN (ref. 1). The bacteria extend these pili to attach to surfaces, and retract them to pull the bacteria towards the point of attachment, mediating irreversible attachment or surface associated twitching motility^2^. The T4aP system is homologous to the type IVb pilus system, the type II secretion system, archaeal flagella (archaella), and bacterial competence systems^3^,^4^. Collectively, these machines can be identified in every major phylum of prokaryotic life^5^.

Despite the importance of the T4aP and related systems, little is known about how the motors of these machines work. It is thought that the energy for pilus extension and retraction is provided by hexameric ATPases in the cytoplasm, known as PilT-like ATPases. The typical T4aP system has two PilT-like ATPases: PilB and PilT. PilB is thought to promote the polymerization of PilA monomers into a long helical filament; this polymerization leads to pilus extension^2^. Conversely, PilT is thought to facilitate pilus retraction by depolymerizing the PilA filament^6^. However, the manner in which PilB/PilT contributes to PilA polymerization is unknown, as the only cytoplasmic region of PilA is a short leader sequence that is cleaved at the inner face of the cytoplasmic membrane before polymerization^7^. Pull-down experiments that indicate PilB interacts with the N-terminal domain of PilC (PilC^NTD^), lead us to predict that PilC might bridge the gap between PilA and PilB/PilT by binding PilA in the inner membrane and the ATPases in the cytoplasm^8^. This prediction is consistent with a recent electron cryotomography-derived model of the T4aP machinery^9^. In this study it was hypothesised that PilB and PilT might function by rotating PilC to stimulate PilA polymerization or depolymerization^9^. In keeping with this model, PilC was recently shown to interact directly with and stimulate PilB activitiy^10^.

Other well-characterized examples of PilT-like ATPases include GspE of the type II secretion system, FlaI of the archaeal flagellar system, and VirB11 of the type IV secretion system^11^. PilT-like ATPases are a family of the Additional Strand Catalytic ‘E' (ASCE) superfamily of ATPases, and as such are related to—but phylogenetically distinct from—FtsK-like ATPases and AAA+ ATPases^12^. Enzymes in the ASCE superfamily contain a Walker A motif and Walker B motif used for binding the phosphates of ATP and coordinating a magnesium ion, respectively^13^. The Walker B motif of PilT-like ATPases is atypical, as the acidic residue essential for magnesium coordination is replaced with glycine^14^. Immediately following the Walker B motif is a glutamate involved in coordinating water for hydrolysis of the γ-phosphate of ATP^15^. PilT-like ATPases also contain conserved histidines in a unique HIS-box motif, and conserved acidic residues in a unique ASP-box motif^16^. While mutations to these motifs in PilT-like ATPases disrupt ATPase activity and *in vivo* function^14^,^16^,^17^, the specific functions of the atypical Walker B, HIS-box and ASP-box motifs are not understood, leading to an incomplete picture for how ATP hydrolysis may power T4aP-like systems.

The available structures of PilT-like ATPases do not unanimously suggest how these enzymes might turn PilC to power pilus extension or retraction, in part due to the heterogeneity in symmetry of the PilT-like hexameric ATPase structures^17^,^18^,^19^,^20^,^21^. For instance, it is difficult to envision how a symmetric C6 hexamer might rotate PilC unless all six chains simultaneously bound and catalysed ATP, as proposed for the SV40 large T-antigen^22^. Most ASCE ATPases are thought to use a rotary mechanism for ATP turnover, operating with either no symmetry, C2 symmetry, or C3 symmetry^15^. However, the C2 symmetric structures of PilT and GspE, and the C3 symmetric structure of FlaI failed to suggest a model for ATP binding and turnover, because the resolution or nucleotide occupancy of these structures was not sufficient to unambiguously identify bound nucleotides^18^,^20^,^21^. The C2 symmetric structures of FlaI and the C3 symmetric structure of archaeal GspE2 are of sufficient resolution to identify bound nucleotides, but during crystallization, these proteins were saturated with ATP or AMP-PNP, respectively, and as a result, all six sites are occupied by the same nucleotide^17^,^21^. With all sites occupied by the same nucleotide, it is difficult to conclude with certainty which sites in a hexamer have high/low affinity for ATP and/or ADP.

Here we determined crystal structures of PilB from *Geobacter metallireducens* under non-saturating nucleotide conditions at 3.4 and 2.3 Å resolution. The differences in nucleotide binding between chains allowed us to deduce a general mechanism for ATP binding and turnover in PilT-like ATPases.

## Results

### Phylogenetic analysis highlights highly conserved residues

**Figure 1:**
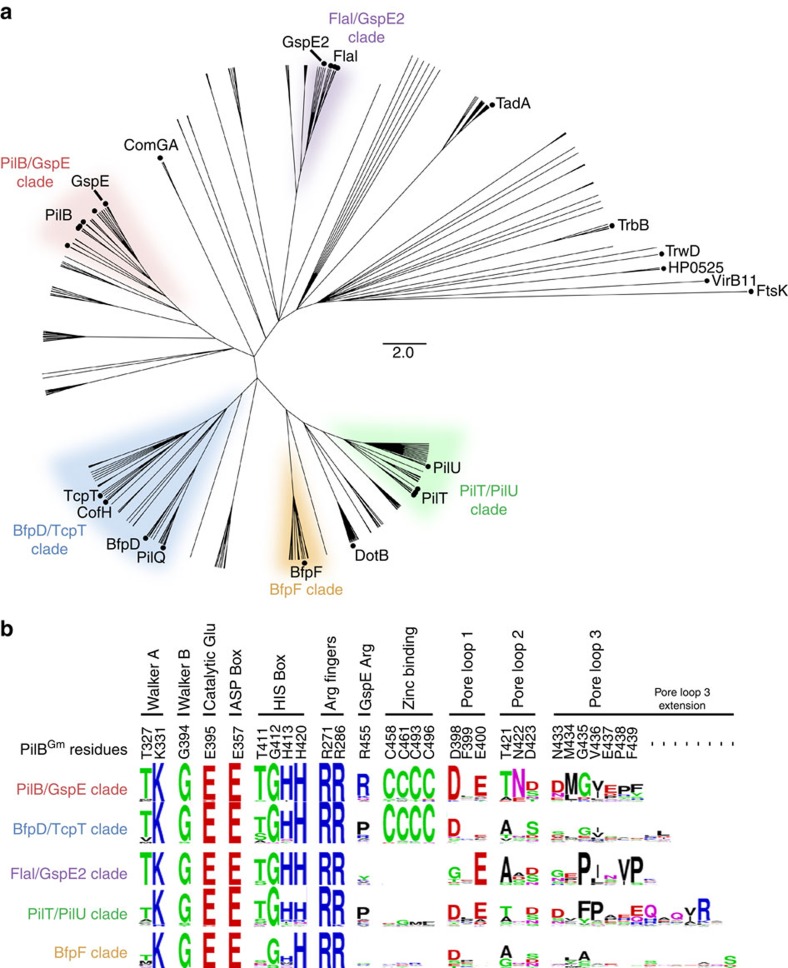
Identification and functional prediction of conserved residues in PilB. (**a**) Overview of the phylogenetic analysis of PilT-like ATPase family members. Sequences from model systems are identified with a black circle and labelled. There are multiple circles for PilB, PilT and FlaI reflecting that there are multiple model systems from different species for these proteins. For a detailed view of the phylogenetic tree including the identity of branches not labelled here see Supplementary Fig. 1. Only branches with a >85% bootstrap value are shown (1,000 bootstraps). FtsK was used as an out-group. Protein sub-families, labelled as clades, are given a unique colour for clarity and to stratify the sequences for further analysis. (**b**) Sequence logo representation of conserved residues stratified based on the clade definitions above. Each dash indicates that there is no corresponding residue in PilB from *G. metallireducens*.

A phylogenetic approach was used to organize the members of the PilT-like ATPases into sub-families to establish important residues shared between sub-families. The sequences of PilT-like ATPases were aligned and a phylogenetic tree was created as described in the Experimental Procedures (Fig. 1a) As expected, proteins with similar functions clustered together. PilT and PilU are tightly clustered, and cluster near the BfpF retraction ATPase. PilB and GspE also clustered together. BfpD, TcpT, PilQ, CofH and LngH extension ATPases from the type IVb pilus clustered together. Archaeal GspE2 clustered closely with FlaI. These results are similar to those obtained previously^11^.

The conservation of important residues in each sub-family or clade was plotted as sequence logos, revealing residues with complete conservation in this family (Fig. 1b and Supplementary Fig. 1): residues in the Walker A and B motifs, the catalytic glutamate, two arginine fingers and a glutamate of the ASP box. A histidine of the HIS box was also highly conserved. We identified residues conserved within sub-families, but not between sub-families: for instance, a tetra-cysteine zinc-binding motif, characterized in GspE and PilB^23^,^24^, was conserved in the PilB/GspE and BfpD/TcpT clades, but not in the FlaI/GspE2, PilT/PilU and BfpF clades. Interestingly, an arginine previously shown to mediate an inter-chain contact in hexameric GspE^20^ was conserved throughout the PilB/GspE clade. This arginine is not conserved in the other clades.

### PilB is an elongated hexamer with C2 symmetry

The phylogenetic clustering of GspE with PilB, as well as the conservation of an arginine used to mediate an inter-chain contact, suggested that PilB might form a hexamer similar to that of GspE (PDB 4KSR). After screening several PilB constructs, we found that PilB from *G. metallireducens* (PilB_Gm_) formed crystals that diffracted anisotropically to 3.4 Å in one dimension and 3.9 Å in the other two dimensions. Screening of many different crystals and crystal conditions revealed the anisotropy to be a consistent phenomenon. The structure of PilB was solved by molecular replacement using GspE as the search model, and we were able to build residues 181–568 in each chain. These residues encompass the second N-terminal domain (N2D), and the C-terminal domain (CTD), residues 181–288 and 296–568, respectively. A flexible linker, residues 289–295, connects the two domains. Despite starting with full-length PilB_Gm_, the first N-terminal domain (N1D) of PilB_Gm_ could not be built into the electron density. Removing these residues from the construct did not disrupt crystallization or improve diffraction quality.

**Figure 2:**
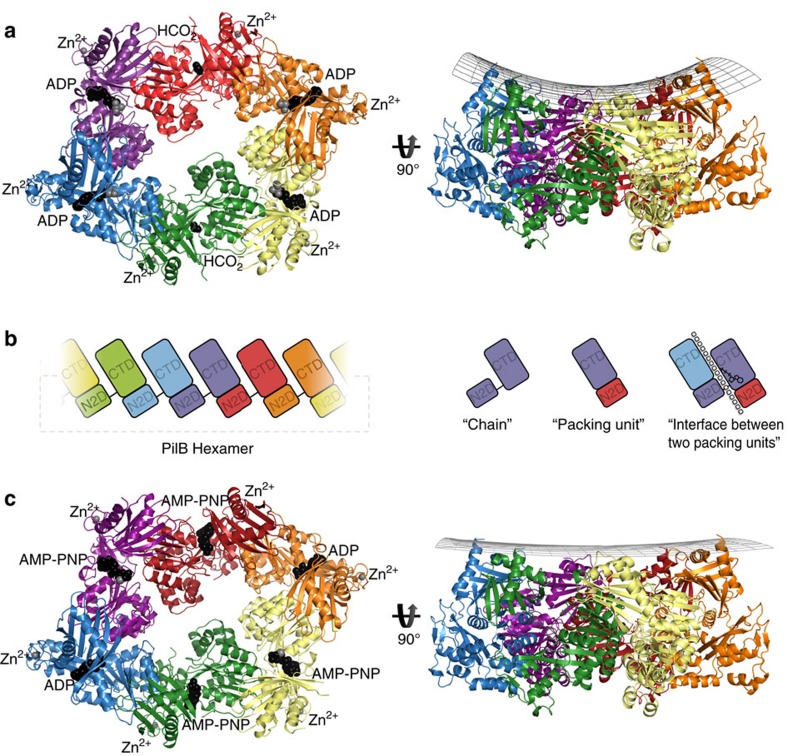
Structure of PilB. (**a**) PilB:ADP hexamer, with each chain indicated in a different colour. ADP and formate are shown as black spheres, while magnesium and zinc are shown as grey spheres. A side view is shown with a grid drawn to emphasize the saddle-like shape. (**b**) Cartoon block illustrations of the PilB hexamer demonstrating the packing units observed, as well as defining the terminology used herein. In the cartoon on the far right, the interface between packing units is represented by a dashed line and the location of the ATP shown in stick representation. (**c**) PilB:AMP-PNP hexamer, with each chain coloured as in **a**. AMP-PNP and ADP are shown as black spheres, while magnesium and zinc are shown as grey spheres. A side view is shown with a grid drawn to emphasize the planar shape.

Three chains could be built in the asymmetric unit, and an elongated hexamer was identified in the crystal packing (Fig. 2a). Consistent with our phylogenetic analysis, the PilB_Gm_ hexamer was most similar to the elongated GspE hexamer (RMSD^Cα^ 2.7 Å per chain, RMSD^Cα^ 5.9 Å per entire hexamer). There is anomalous signal in the predicted tetra-cysteine zinc-binding sites, so zinc was also modelled into these motifs in each chain. There is density in the predicted ATP binding sites of all six chains. For two sites, the density was too small for a nucleotide. Instead, formate, which was present in the crystallization buffer, was modelled. In four sites, ADP and magnesium best fit the electron density, even though no exogenous nucleotide was added during crystallization. These nucleotides must have been pulled down from the cytoplasm of *E. coli* during purification. Herein this structure will be referred to as PilB:ADP.

The principle inter-chain contact, with a buried surface area of ∼1,600 Å, results from an interaction between the N2D and the CTD of adjacent chains. This interaction links the N2D of one chain to the CTD of an adjacent chain as a single packing or construction unit, a nomenclature used previously for GspE^20^. The aforementioned flexible linker between the N2D of one packing unit and CTD of an adjacent packing unit connects two packing units. This configuration creates a hexamer of packing units resembling six beads on a string (Fig. 2b). Henceforth, where relevant, we refer to these packing units instead of individual chains, as this enables us to better describe the intra- and inter-chain conformational changes that correlate with nucleotide binding.

### The pore of the PilB hexamer is conserved and negatively charged

**Figure 3:**
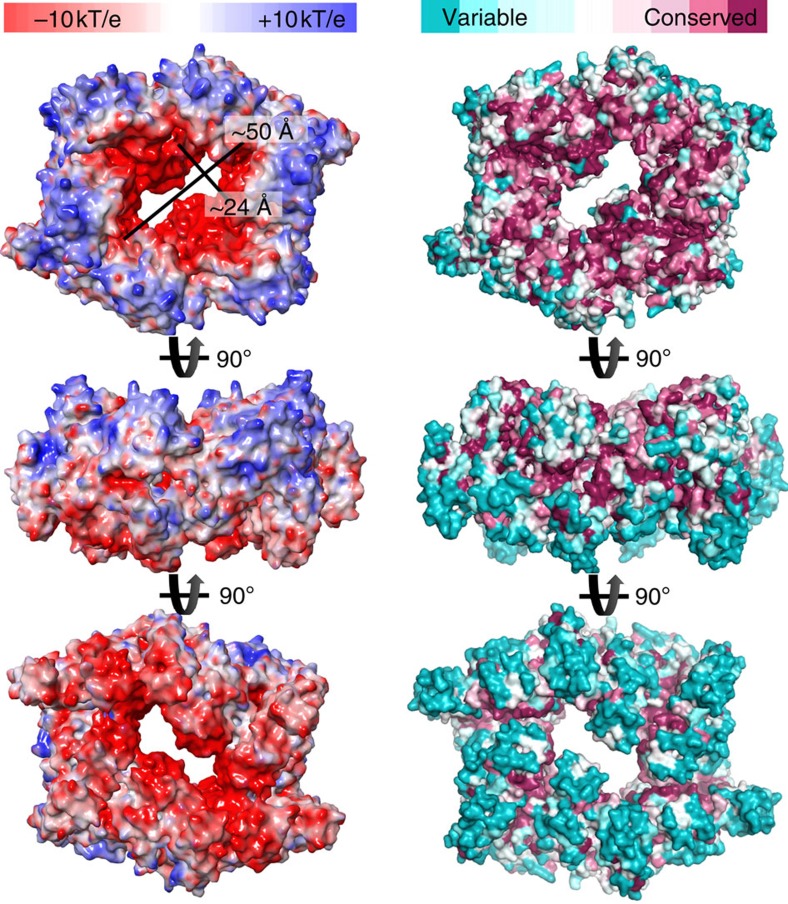
Surface representations of PilB from *G. metallireducens*. (Left) The electrostatic surface of PilB calculated with missing side chains added in the most likely rotamer via Maestro (version 10.5, Schrödinger). (Right) The phylogenetic conservation of PilB residues mapped onto the surface of PilB using the ConSurf server^51^.

Assessing the phylogenetic conservation and surface electrostatics of PilB:ADP revealed that there is a conserved and negative surface in the pore of the PilB:ADP hexamer (Fig. 3). At its widest, the pore is ∼50 Å; at its narrowest, ∼10 Å. The elongated shape of the structure gives the pore the appearance of two ∼24 Å diameter sub-pores. The perimeter of the pore is less conserved and positively charged. As it is anticipated that PilC binds to the pore of PilB^8^, we extended the aforementioned phylogenetic analysis to residues in the pore of PilB_Gm_ to reveal patterns of conservation between different PilT-like ATPases. Residues in pore loops 2 and 3 are conserved within sub-families but not between sub-families, while D398 or E400 of pore loop 1 is conserved across most PilT-like ATPases (Fig. 1b). Of note, there are six and seven additional residues in pore loop 3 of the PilT/PilU clade and BfpF clade, respectively.

### Nucleotide binding correlates with conformational differences

**Figure 4:**
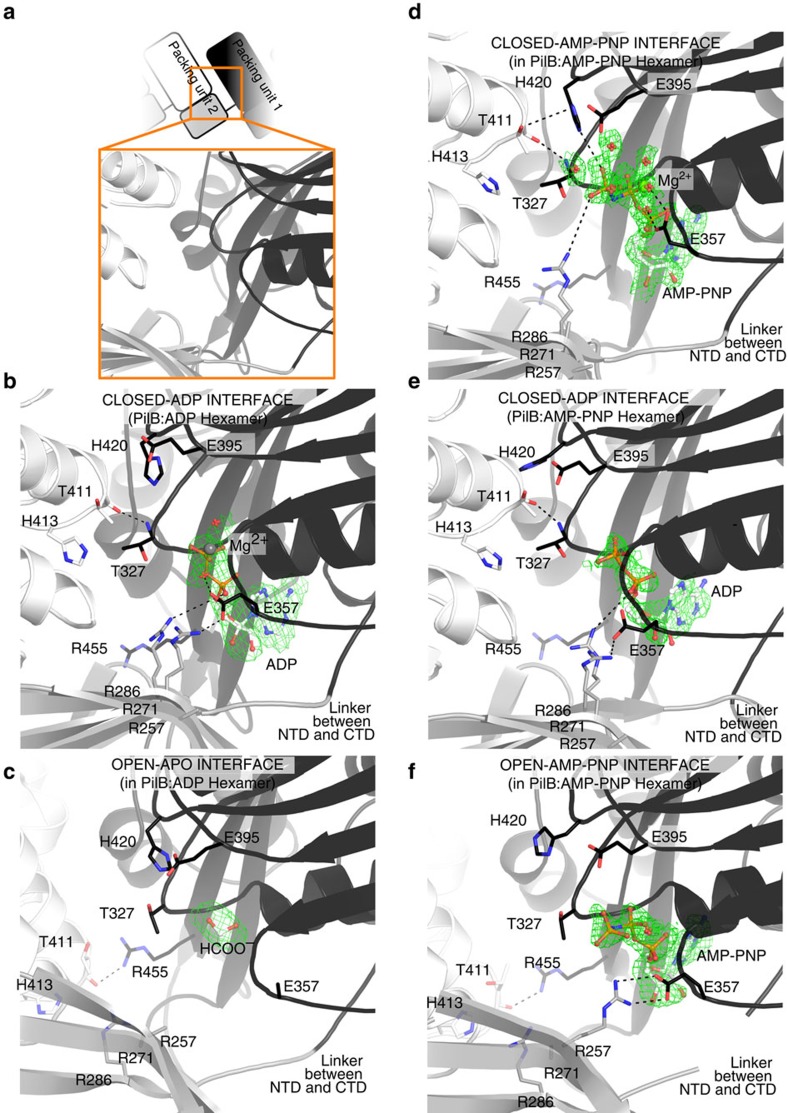
ATP binding sites of PilB. Direct polar contacts are shown as dashed lines. Magnesium is shown as a grey sphere. The green mesh represents the Feature Enhanced Map computed by PHENIX-FEM^45^ contoured at 2.0σ. (**a**) Cartoon clarifying the identity of domains in the following sub-Figures. The cartoon mirrors Fig. 2b. (**b**) Nucleotide binding site in the closed-ADP interface from PilB:ADP. (**c**) Nucleotide binding site in the open-APO interface from PilB:ADP. (**d**) Nucleotide binding site in the closed-AMP-PNP interface from PilB:AMP-PNP. (**e**) Nucleotide binding site in the closed-ADP interface from PilB:AMP-PNP. (**f**) Nucleotide binding site in the open-AMP-PNP interface from PilB:AMP-PNP.

In PilB:ADP, ADP is bound between two packing units. One packing unit, packing unit 1, binds ADP via the Walker A motif with magnesium coordinated by E357 of the ASP box. E357 of the ASP box assumes the role of magnesium coordination typically played by the Walker B motif in other ASCE ATPases accounting for the atypical Walker B motif seen in PilT-like ATPases. The adjacent packing unit, packing unit 2, binds the phosphates of ADP via R286, and binds E357 of the ASP box via R271 (Fig. 4a,b). At this interface, the backbone of T327 from packing unit 1 contacts the backbone of T411 in packing unit 2, while the side chain of T411 is sandwiched between H413 and H420 of the HIS box.

The PilB:ADP structure also contains an apo interface between two packing units, where no nucleotide is present. At this interface, the orientation of the packing units does not facilitate the binding of T327 to T411 but instead, the side chain of R455 of packing unit 1 binds the backbone of T411 of packing unit 2, while the flexible linker plays a more active role in tethering packing units together (Fig. 4c).

Comparing this apo interface to the ADP-bound interface suggests that two packing units close by ∼60° to grasp the nucleotide^25^ (Supplementary Fig. 2a). Henceforth, the ADP-bound and apo interfaces will be referred to as the closed-ADP and open-APO interfaces, respectively. The absence of nucleotide in the open interface suggests that this site has a relatively low affinity for ADP compared with the closed interfaces. Furthermore, the presence of ADP at the closed-ADP interfaces indicates that PilB_Gm_ does not immediately release ADP following ATP catalysis. ADP release from the closed-ADP interface may be contingent on a separate event in an adjacent packing unit, such as ATP binding to the open-APO interface.

The closed-ADP interface has helical character (Supplementary Fig. 3), and thus six packing units connected by such interfaces would not create a hexamer, but rather a helix with approximately −12 Å rise and 65° twist. Likewise, the open-APO interface also has helical characteristics, but with ∼+24 Å rise and 76° twist. With four closed-ADP interfaces, and two open-APO interfaces, the net rise equals zero; and thus six packing units form a ring instead of a helix. This scenario implies conformational restraints on the hexamer to maintain a closed ring: every two closed-ADP interfaces require an open interface to counter the change in rise. This pattern of one open interface for every two closed-ADP interfaces gives the hexamer its elongated appearance. The net twist applied by these interfaces is greater than 360°, so in addition to being elongated, the hexameric ring puckers, similar to the boat conformation of cyclohexanes (Fig. 2a).

### Structure determination of PilB bound to an ATP analogue

To trap PilB_Gm_ in a conformation similar to the ATP-bound state, PilB_Gm_ was incubated with AMP-PNP, a non-hydrolysable ATP analogue, under conditions similar to those that facilitated crystallization of PilB:ADP. Crystals formed when AMP-PNP was added to a final concentration of 50 μM to a 120 μM solution of PilB_Gm_—a ratio of 5:12 nucleotides to ATP binding sites. The crystals diffracted to 2.3 Å in all three dimensions. The structure was solved by molecular replacement, and again—despite starting with full-length PilB_Gm_—the N1D of PilB_Gm_ could not be built into the electron density. Six chains could be built in the asymmetric unit, which together formed an elongated hexamer similar to the previously identified PilB_Gm_ hexamer (Fig. 2c). Henceforth, this AMP-PNP bound hexamer is referred to as PilB:AMP-PNP.

As in the PilB:ADP hexamer, there was density in the predicted ATP binding sites of all six packing units of the PilB:AMP-PNP structure. However, there were significant differences in the PilB:AMP-PNP hexamer. Between two packing units, AMP-PNP, magnesium, and several highly ordered waters best fit the electron density. Between two other packing units, ADP with ∼50% occupancy best fit the electron density. Between the final two packing units, AMP-PNP with ∼70% occupancy best fit the electron density.

### AMP-PNP binding induces a distinct closed interface

The overall structure of PilB:AMP-PNP is similar to that of PilB:ADP, in that it has four closed and two open packing interfaces. PilB:AMP-PNP is distinct from PilB:ADP in that only two closed interfaces have ADP bound, and they are bound with partial occupancy. The other two closed interfaces are bound to AMP-PNP with full occupancy, henceforth referred to as the closed-AMP-PNP interface. AMP-PNP is also bound with partial occupancy in the two open interfaces and will be referred to herein as the open-AMP-PNP interface. This occupancy pattern suggests that the two closed-AMP-PNP interfaces have the highest affinity for AMP-PNP, and by extension ATP, while the open interfaces have a moderate affinity for ATP, and the closed-ADP interfaces have low affinity for ATP. The partial occupancy of ADP at the closed-ADP interface of PilB:AMP-PNP is consistent with limited nucleotide binding to the open interface, triggering ADP release from the adjacent closed-ADP interface.

The polar contacts created by the closed-ADP and open-AMP-PNP interfaces in the PilB:AMP-PNP hexamer were very similar to the closed-ADP and open-APO interfaces in the PilB:ADP hexamer, respectively (Fig. 4e,f). However, in the open-AMP-PNP interface, E357 of packing unit 1 forms a salt bridge with R257 of packing unit 2. This interaction did not occur in the open-APO interface of PilB:ADP.

The closed-AMP-PNP interface is also similar to that of the closed-ADP interface of PilB:ADP. The backbone of T327 from packing unit 1 still contacts the backbone of T411 from packing unit 2. However, H420 of the HIS box coordinates the γ-phosphate of AMP-PNP and the side chain of T411 from packing unit 2, suggesting that the HIS box plays a role in mediating the inter-chain cooperativity of ATP catalysis. In addition, E357 is reoriented to coordinate magnesium, which contacts the β- and γ-phosphate. In this position, E357 from packing unit 1 cannot bind R271 from packing unit 2. Nearby, R286 coordinates the γ-phosphate of the AMP-PNP instead of coordinating the α-phosphate of ADP (Fig. 4d). In this way, R286 and R271 in packing unit 2 sense the presence of the γ-phosphate bound to packing unit 1, and twist the closed-AMP-PNP interface by 8° relative to the closed-ADP interface.

The helical rise and twist created by the closed-AMP-PNP interface is approximately −12 Å and 62°, respectively, which is accommodated by the open interface that applies approximately +24 Å rise and 72° twist. The closed-ADP interface still has approximately −12 Å rise and 65° twist. The net decrease in twist reduces the ring puckering relative to the boat shape of PilB:ADP such that the PilB:AMP-PNP ring appears more planar (Fig. 2c).

### Heterogeneous nucleotide binding indicates catalytic mechanism

Altogether, the PilB:ADP and PilB:AMP-PNP structures suggest the relative affinities of ATP and ADP for the different interfaces observed in the PilB_Gm_ hexamer. The open interface has a low affinity for ADP and a moderate affinity for ATP. With the N2Ds facing the viewer, the closed interface immediately clockwise of the open interface has a high affinity for ADP and a low affinity for ATP. The next closed interface has a high affinity for ATP and ADP. This provides evidence for the mechanism of ATP turnover. Starting with the PilB:ADP hexamer—PilB_Gm_ bound to four ADP molecules—added ATP would bind to the two open interfaces, since the closed interfaces are occupied with ADP. ATP binding to the open interface would result in the closure of this interface, and because of the aforementioned rise restraints on the hexamer, this would trigger opening of two closed-ADP interfaces, and the release of ADP. We modelled this structural change in PilB_Gm_ with an interpolated trajectory of the PilB:ADP structure to the PilB:AMP-PNP structure (Supplementary Movie 1). The model reflects the expectation that ATP binding induces closure of the two open interfaces, while opening two of the closed interfaces. The location of the closed-AMP-PNP and closed-ADP interfaces in the PilB:AMP-PNP hexamer demonstrates that there is directionality to the release of ADP. ADP is released from the closed interfaces immediately clockwise from the open interfaces that bind ATP. Finally, PilB_Gm_ would be reset after ATP catalysis, yielding PilB_Gm_ bound to four ADP molecules.

**Figure 5:**
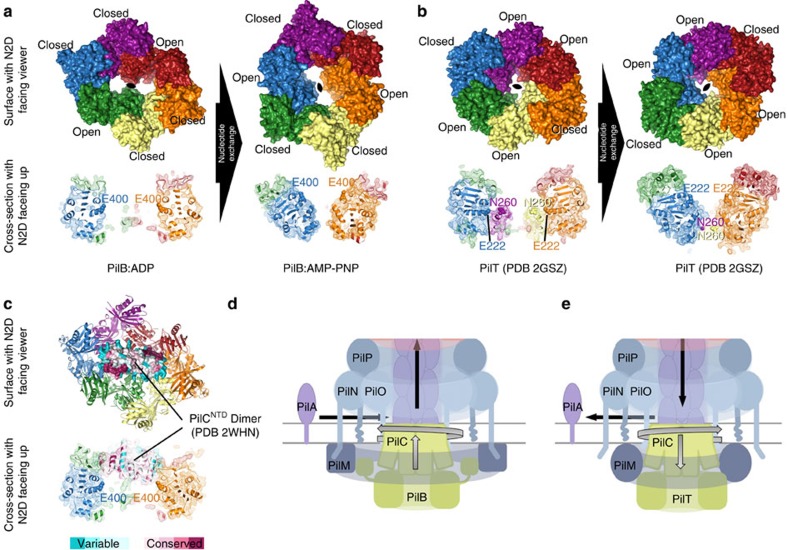
Modeling the movements and functions of PilB and PilT ATPases. (**a**) The PilB:ADP hexamer was aligning with the PilB:AMP-PNP hexamer to suggest the movements that may occur as two closed packing units open and two open packing units close during nucleotide exchange. Chains are coloured as in Fig. 2. (Bottom) A cross-section through the centre of PilB is shown with E400 displayed as spheres. (**b**) The PilT hexamer (PDB 2GSZ) was aligned with the same PilT hexamer rotated by one packing unit to suggest the movements that may occur as two closed packing units open and two open packing units close during nucleotide exchange. (Bottom) A cross-section through the centre of PilT is shown with E222 (the equivalent of E400 in PilB) and N260 from the midpoint of the extension on pore loop 3 (there is no equivalent in PilB) displayed as spheres. (**c**) The PilC^NTD^ dimer (PDB 2WHN) was manually placed in the two sub-pores of PilB, and the phylogenetic conservation of PilC residues were mapped onto the surface using the ConSurf server^51^. (Bottom) A cross-section through the centre of PilB is shown with E400 displayed as spheres. The model is similar to the mode of PilC binding to PilB we proposed previously^8^. (**d**) Working model for the molecular mechanism of the PilB motor. We propose that PilC is thrusted by PilB upwards towards the membrane, allowed to fall back, and rotated in 60° increments to facilitate helical PilA polymerization. (**e**) Working model for the molecular mechanism of the PilT motor. We propose that PilC is wrenched downward by PilT towards the cytoplasm, allowed to relax upwards, and rotated in 60° increments in the opposite direction of PilB to facilitate helical PilA depolymerization. The model presented in panels D and E refines our previous model that described how PilM-PilN interactions modulate PilB/PilT associations^27^.

To model the structural changes in PilB_Gm_ that may occur as ATP is catalysed to ADP, we created an interpolated trajectory of the PilB:AMP-PNP structure to the PilB:ADP structure. The model reflects the expectation that ATP catalysis does not directly cause interface closure or opening. In this model, one can visualize that ATP catalysis may cause the relatively planar PilB_Gm_ hexamer to adopt a saddle shape (Supplementary Movie 2). Merging these models into an complete model of ATP binding, catalysis, and release reveals that ATP binding causes two motions, perpendicular to one another, in the PilB_Gm_ hexamer: a clockwise rotation of the sub-pores about the axis perpendicular to the plane of the hexamer, and a rotation of the two packing units that went from being closed to open about the axis parallel to the plane of the hexamer (Fig. 5a; Supplementary Movie 3). With the N2Ds facing the viewer, the sub-pore rotation is clockwise and the packing units thrust towards the viewer. The conserved pore residue E400 on the thrusting packing units moves towards the viewer by ∼13 Å.

### PilT produces opposite pore movements relative to PilB

We next compared the PilB_Gm_ structures to that of the previously published C2 symmetric PilT hexamer^18^. By structural comparison with the interfaces of PilB_Gm_, we identified two closed and four open interfaces in PilT, surrounding a central elongated pore (Supplementary Fig. 2). Interestingly, PilB_Gm_ and PilT appear to have an enantiomeric arrangement of open and closed interfaces (Fig. 5b).

On the basis of direction of ATP turnover in PilB_Gm_, we modelled the structural changes that may occur as ATP is turned over by PilT. Similar to the animated PilB_Gm_ model, the animated PilT model reflects the expectation that nucleotide exchange induces closure of two open interfaces and opening of two closed interfaces. As in PilB_Gm_, this exchange causes two motions perpendicular to one another in the PilT hexamer: a rotation of the elongated pore, and a rotation of the two packing units that went from being closed to open (Fig. 5c; Supplementary Movie 4). Despite assuming the same direction of ATP turnover as PilB_Gm_, with the N2D facing the viewer, the rotation of the PilT pore is counterclockwise, opposite that of PilB. Although there is a rotation of the two packing units that went from being closed to open, in PilT these packing units have limited access to the pore due to steric hindrance from the extension of residues on pore loop 3 from another packing unit in the hexamer. Notably, during rotation the residues in this extension pull away from the viewer by ∼19 Å. In other words, if we assume the same direction of ATP turnover in PilB_Gm_ and PilT, opposite pore rotation and perpendicular movements are generated as a consequence of two unique adaptions in PilT: the enantiomeric arrangement of open and closed interfaces, and an extension of residues on pore loop 3.

## Discussion

The heterogeneous nucleotide binding observed in our PilB_Gm_ structures reveals a correlation between nucleotide binding and inter-chain interfaces that enabled us to define with confidence the direction of ATP turnover, and deduce the conformational changes that occur during nucleotide exchange. The closed-AMP-PNP interface of the PilB_Gm_:AMP-PNP structure is the only interface with the catalytic glutamate, E395, poised for hydrolysis, suggesting that this is the site of ATP catalysis. In addition, the vacancy and partial occupancy of nucleotide in the open interfaces of the PilB_Gm_:ADP structure and PilB_Gm_:AMP-PNP structure, respectively, indicate that this site is used for ADP/ATP exchange. With this knowledge, we built a model for PilB movements and then expanded our analysis to the related ATPase, PilT.

We identified pore residues that are highly conserved in PilT-like ATPases. As previously hypothesized, PilC^NTD^ is expected to bind in the central pore of PilB^8^, and thus these residues may be important for interaction with PilC. A crystal structure of PilC^NTD^ from *T. thermophilus* was solved as an asymmetric dimer^26^. The diameter of each PilC^NTD^ chain is approximately 23 Å, and thus a PilC^NTD^ dimer would fit snugly in the two ∼24 Å sub-pores of PilB (Fig. 5c). The C-terminal domain of PilC (PilC^CTD^) is orthologous and a similar length to PilC^NTD^, and thus it is also plausible that the PilC^NTD^ could fit in one sub-pore and PilC^CTD^ in the other. Examining the phylogenetic conservation of the PilC^NTD^ dimer in the context of our model of PilB hexamer movements revealed a patch of conserved residues in PilC^NTD^ adjacent to the packing units that we predict thrust through the pore. With this in mind, we propose that PilB functions by thrusting a PilC monomer or dimer upwards into the inner membrane. As two ATP molecules are synchronously hydrolysed in the PilB hexamer, we predict that the rotary motion of the PilB sub-pores would turn PilC clockwise in 60° increments. By the end of the 60° rotation, PilC will no longer be oriented to bind the packing units that thrust through the pore. Thus, we propose that PilC is pushed upwards in the membrane, allowed to fall back, and rotated by 60° in a single motion (Fig. 5d; Supplementary Movie 5). There are several lines of evidence indicating that PilC binds directly to the pilin subunit, PilA^9^,^27^,^28^, and thus these motions could facilitate PilA extraction from the membrane and insertion into the helical pilus. We envision PilB as the cylinder, spring, and trigger of a revolver, which rotationally aligns the hammer (PilC) with the bullets (PilA) to consecutively fire. If a pilin subunit is inserted with every thrust, the resulting pilus filament would be a right-handed helix with a 60° twist. This is consistent with reports in the literature that suggest the pilus is a right-handed helical fibre with a range of twists (60–100°) within and between systems and species^29^,^30^,^31^,^32^,^33^, and thus we predict that the pilus itself facilitates further twisting upon assembly—as predicted by molecular dynamic simulations^29^.

Applying similar transformations to the available structure of C2 symmetric PilT^18^ allowed us to model the structural changes that may occur as nucleotide is exchanged. Despite applying the same direction of opening and closing interfaces, the rotation of the PilT pore would be opposite that of PilB. This is the direct consequence of the enantiomeric arrangement of open and closed interfaces in PilB and PilT (Supplementary Fig. 5). Furthermore, a conserved extension on pore loop 3 pulls laterally through the pore in the opposite direction of the thrusting motion generated in PilB. We predict that PilC binds to the extension on pore loop 3, and propose that PilT acts as an anti-PilB: rotating PilC in the direction opposite that of PilB and pulling PilC towards the cytoplasm to facilitate PilA depolymerization (Fig. 5e and Supplementary Movie 5).

A C2 symmetric structure of *Thermus thermophilus* PilB (PilB_Tt_) at 2.65 Å resolution was recently published^34^. Using the packing unit nomenclature PilB_Tt,_ similar to PilB_Gm_, has four closed and two open interfaces between packing units (Supplementary Fig. 2). In contrast to the structures of PilB_Gm_, the nucleotide binding sites in the PilB_Tt_ hexamer were modelled to be homogeneously saturated with the ATP analogue, ATPγS. The conformation of ATPγS in the four closed interfaces of PilB_Tt_ does not match that of AMP-PNP in PilB_Gm_. The resolution and quality of the electron density of the 2.3 Å resolution PilB_Gm_ structure and the 2.0 Å resolution structure of ADP-Mg^2+^ bound to FlaI^21^ were sufficient to enable the octahedral water coordination of the magnesium to be modelled, permitting unequivocal differentiation between the γ-phosphate and Mg^2+^ in these structures. Comparison of these structures with PilB_Tt_ suggests that the γ-phosphate of ATPγS in the PilB_Tt_ structure occupies the position of the Mg^2+^ (Supplementary Fig. 4). Therefore, an alternative explanation may be that ATPγS was hydrolysed, as observed during crystallization of the PilB orthologue, PilT^18^. The PilB_Tt_ model with ATPγS led Mancl *et al*.^34^ to propose a direction of ATP turnover that is the opposite to the direction proposed herein, i.e., that the PilB_Tt_ pore rotates counterclockwise.

While our proposed PilB and PilT mechanisms rely on C2 symmetry with restricted open and closed interfaces, we cannot omit the possibility of alternative interfaces not characterized here, as there are models of FlaI and archaeal GspE2 with C3 symmetry. That FlaI and archaeal GspE2 would form similar hexamers is not surprising, given their clustering in our phylogenetic analysis. It is interesting to note, however, that unlike other PilT-like ATPases, FlaI could be responsible for two rotary functions: turning the PilC homologue FlaJ to power assembly as outlined for PilB^35^, and then spinning the assembled archaeal flagellum. If the C2 rotary model is responsible for the former, it is tempting to speculate that the unique C3 symmetry of FlaI could reflect a separate functional state involved in turning the archaeal flagellum, once assembly of the filament is complete.

An assumption implicit in the C2 rotary model is the orientation of PilB in relation to the inner membrane and the pilus. We based this assumption on the electrostatic surface properties of PilB; the positive face on the PilB hexamers may correspond to an interface used to bind the inner membrane. Indeed, electron cryotomography studies show PilB adjacent to the cytoplasmic face of the inner membrane^9^, and the PilB-like ATPase, GspE, has increased ATPase activity in the presence of cardiolipins, suggesting that it binds directly to the inner membrane^36^.

We noted that PilB:ADP adopted a saddle shape, while the AMP-PNP bound conformation was relatively planar. This architecture implies that upon ATP catalysis, the inner membrane may be deformed through its interaction with PilB, and/or that the inner membrane applies a strain on these saddle-shaped hexamers. This strain would then be released as the hexamer bound more ATP and thrust PilC, adopting a planar state. Decoupling the energy-generating step from the energy-using step using strain is a common strategy used in macroscopic biology to amplify force^37^,^38^. Thus, it is possible that PilB temporarily stores strain to thrust PilC with amplified force. If PilT and PilB act in similar manners, it would help explain the extraordinary mechanics of pilus extension and retraction.

## Methods

### Expression and purification of PilB

PilB from *G. metallireducens* was PCR amplified from genomic DNA using primers P96 (TATATATAGCTAGCATGCAGGCCAGCAGACTG) and P97 (TATATATAGGATCCTTAATCGTCGGCCACGGTG), digested with NheI and BamHI, and cloned into pET28a with a thrombin-cleavable hexahistadine tag to create pET28a:PilB^Gm^. The fidelity of the sequence was verified by TCAG sequencing facilities (SickKids, Canada). *Escherichia coli* BL21-CodonPlus cells (F^−^ *ompT hsdS* (rB^−^ mB^−^) dcm^+^ Tet^r^ galλ (DE3) endA [*argU proL* Cam^r^]; Stratagene, USA) transformed with pET28a:PilB^Gm^, grown in 4 l of lysogeny broth (LB) with 100 μg ml^–1^ kanamycin at 37 °C to an A600 of 0.5–0.6. Protein expression was induced by the addition of isopropyl-D-1-thiogalactopyranoside (IPTG) to a final concentration of 1 mM, and the cells were grown for 16 h at 18 °C. Cells were pelleted by centrifugation at 9,000 *g* for 15 min. Cell pellets were subsequently resuspended in 40 ml binding buffer (50 mM Tris pH 7.5, 150 mM NaCl and 50 mM imidazole), lysed by passage through an Emulsiflex-c3 high-pressure homogenizer, and the cell debris removed by centrifugation for 60 min at 40,000 *g*. The resulting supernatant was passed over a column containing 5 ml of pre-equilibrated Ni-NTA agarose resin (Life Technologies, USA). The resin was washed with 10 column volumes (CV) of binding buffer and eluted with binding buffer plus 300 mM imidazole. The protein was then further purified by size exclusion chromatography on a HiLoadTM 16/600 SuperdexTM 200 pg column pre-equilibrated with binding buffer without imidazole. Purified proteins were stored at 4 °C for <2 days before use.

### Structure solution

For crystallization, purified PilB was concentrated to 16 mg ml^–1^ at 3,000 *g* in an ultrafiltration device (Millipore). Crystallization conditions were screened using the complete MCSG suite (MCSG 1–4) (Microlytic, USA) using a Gryphon LCP robot (Art Robbins Instruments, USA). Crystal conditions were screened and optimized using vapour diffusion at 20 °C with Art Robbins Instruments Intelli-Plates 96–2 Shallow Well (Hampton Research, USA) with 1 μl protein and 1 μl reservoir solution. For the PilB:AMP-PNP structure, the protein solution also contained 100 μM AMP-PNP. For the PilB:ADP structure the reservoir solution was 13% (w/v) PEG3350, 0.1 M magnesium formate, 0.1 M Tris pH 7.6. For cryoprotection, 2 μl of 1:1 ethylene glycol and reservoir solution was added to the drop containing the crystal for 10 s before vitrification in liquid nitrogen. For the PilB:AMP-PNP structure, the reservoir solution was 11% (w/v) PEG3350, 0.1 M magnesium formate, 0.1 M Tris pH 7.0. For cryoprotection, 2 μl of 1:1 2-methyl-2,4-pentanediol and reservoir solution was added to the drop containing the crystal for 10 s before vitrification in liquid nitrogen. Diffraction data was collected on Beamline 08ID-1 at the Canadian Macromolecular Crystallography Facility (Table 1). The PilB:AMP-PNP data were indexed, scaled, and truncated to 2.3 Å using XDS^39^. The PilB:ADP data were indexed in XDS, then anisotropically truncated to 3.4 Å in one dimension and 3.9 Å in the other two dimensions and scaled using the Diffraction Anisotropy Server^40^. PHENIX-MR^41^ was used solve the structure of PilB by molecular replacement with residues 100–226 and residues 236–500 of GspE (PDB 1P9R) pre-processed by the program Chainsaw^42^. The resulting electron density maps were of high quality and enabled building the PilB proteins manually in COOT^43^. Through iterative rounds of building/remodelling in COOT^43^ and refinement in PHENIX-refine^44^ the structures of the PilB:ADP and PilB:AMP-PNP were built and refined. PilB:ADP was refined with group B-factors, while PilB:AMP-PNP was refined with individual B-factors. Feature enhanced maps^45^ were used to improve the electron density signal of the bound nucleotides. The occupancy of the nucleotides in the PilB:AMP-PNP structure was refined such that all atoms in a nucleotide had the same occupancy. Progress of the refinement in all cases was monitored using Rfree. Model visualization and images were made in The PyMOL Molecular Graphics System, Version 1.8 Schrödinger, LLC. Interpolated trajectory Supplementary Movies were made in Chimera^46^. Supplementary Movie 5 was made in Molecular Flipbook (molecularflipbook.org).

### Phylogenetic analysis

HMMER^47^ was used to identify PilB orthologues. The redundancy of the orthologues was reduced to <40%, while retaining the orthologues from model systems. The remaining sequences were aligned in ClustalOmega^48^, and a Neighbour-Joining tree was built with MEGA using the Poisson substitution model^49^. Sequence logos were generated using WebLogo^50^.

### Data availability

Crystallographic data that support the findings of this study have been deposited in the Protein Data Bank with the accession codes 5TSG and 5TSH. The data that support the findings of this study are available from the corresponding author on request.

## Additional information

**How to cite this article:** McCallum, M. *et al*. The molecular mechanism of the type IVa pilus motors. *Nat. Commun.* **8,** 15091 doi: 10.1038/ncomms15091 (2017).

**Publisher's note**: Springer Nature remains neutral with regard to jurisdictional claims in published maps and institutional affiliations.

## Supplementary Material

Supplementary Information Supplementary Figures

Supplementary Movie 1 Interpolated trajectory between PilB:AMP-PNP and PilB:ADP with two packing units opening and two packing units closing

Supplementary Movie 2 Interpolated trajectory between PilB:ADP and PilB:AMP-PNP.

Supplementary Movie 3 Modelling movements of PilB. Depicted are iterative interpolated trajectories between PilB:ADP and PilB:AMP-PNP and back to PilB:ADP.

Supplementary Movie 4 Modelling movements of PilT.

Supplementary Movie 5 Proposed model of the T4aP motors.

## Figures and Tables

**Table 1:** Data Collection and Refinement Statistics of PilB Structures.

	**PilB:ADP**	**PilB:AMP-PNP**
*Data collection*		
Beamline	08ID-1	08ID-1
Wavelength (Å)	0.97920	0.97920
Space group	*P*3_2_21	*P*1
*a, b, c* (Å)	112.1, 112.1, 299.2	74.3, 100.0, 109.8
α, β, γ (°)	90.0, 90.0, 120.0	113.0, 107.7, 88.9
Resolution (Å)	46–3.40 (3.52–3.40)	48–2.30 (2.38–2.30)
Total reflections	208,303	253,982
Unique reflections	23,642 (375)	120,979 (1,531)
Redundancy	8.8 (10.3)	2.1 (2.1)
Completeness (%)	77 (12)*	96 (95)
Mean *I*/σ*I*	12.7 (2.78)	5.8 (2.0)
*R*_Sym_ (%)†	21 (94)	5.6 (43)
CC* (%)†	100 (96)	100 (88)
*Refinement*		
R_work_/R_free_ (%)‡	27.2/28.9	18.1/22.0
R.m.s.d.		
Bond lengths (Å)	0.007	0.011
Bond angles (deg)	1.42	0.91
Ramachandran plot§		
Total favoured (%)	98	99
Total allowed (%)	100	100
Coordinate error (Å)‖	0.38	0.27
Atoms	8,477	18,174
Protein	8,406	17,443
Water	12	533
Magnesium	2	6
AMP-PNP	0	132
ADP	54	54
Zinc	3	6
Av. B-factors (Å^2^)§	87.7	49.3
Protein	87.9	49.2
Water	36.7	48.2
Ligands	65.6	63.2
PDB	5TSG	5TSH

Values in parentheses correspond to the highest resolution shell.

^*^atypical completeness of the PilB:ADP structure reflects anisotropic truncation.

^†^*R*_Sym_=∑∑|*I*−(*I*)|/∑∑*I*, *R*_Pim_=∑✓(1/(*n*−1)∑|*I*−(*I*)|/∑∑*I*, and CC*=✓(2CC_1/2_/(1+CC_1/2_)) where CC_1/2_ is the Pearson correlation coefficient of two half data sets as described elsewhere^52^.

^‡^*R*_work_=∑||*F*_obs_|−k|*F*_calc_||/|*F*_obs_|, where *F*_obs_ and *F*_calc_ are the observed and calculated structure factors, respectively. *R*_free_ is the sum extended over a subset of reflections (5%) excluded from all stages of the refinement

^§^As calculated using MolProbity^53^.

^‖^Maximum-likelihood based Coordinate Error, as determined by PHENIX^41^.
