# Supplementary material for: The molecular mechanism of the type IVa pilus motors
Source: Nat Commun. 2017 May 5;8:15091. doi: 10.1038/ncomms15091 (PMC5424180; doi:10.1038/ncomms15091)
Supplement: Supplementary Information — Supplementary Figures [file ncomms15091-s1.pdf]

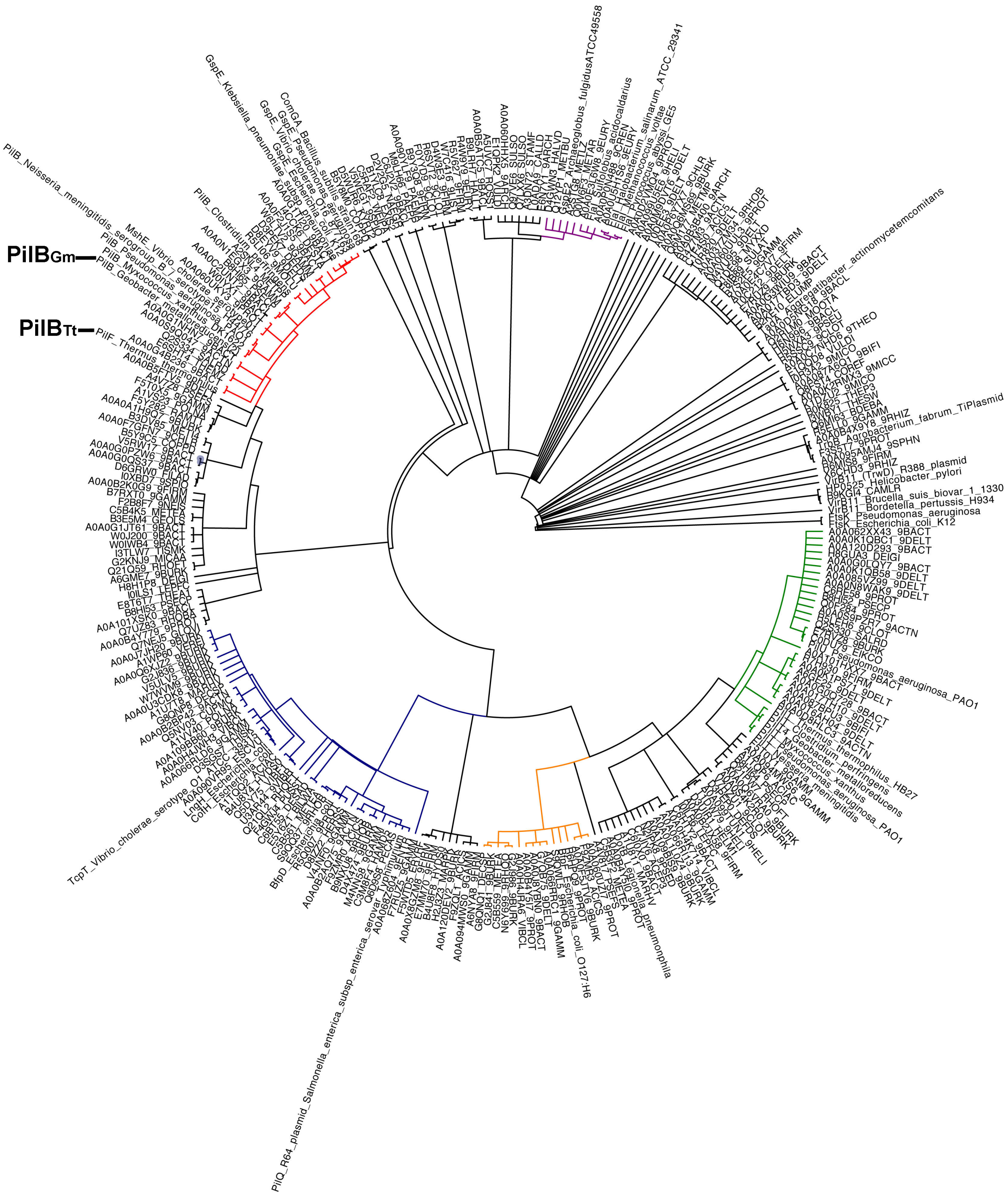

Supplementary Fig. 1. Phylogenetic analysis of PilT-like ATPases. Sequences are labeled with their PFAM identifiers, or with the protein name and species if the sequence is from a model system. Only branches with a >85% bootstrap value are shown (1000 bootstraps). FtsK was used as an out-group. Protein sub-families, or clades, are given a unique color for clarity and to stratify the sequences for further analysis. Red is for the PilB/GspE clade. Purple is for the FlaI/GspE2 clade. Green is for the PilT/PilU clade. Orange is for the BfpF clade. Blue is for the BfpD/TcpT clade. The sequences of $PilB_{Gm}$ and $PilB_{Tt}$ are labeled in bold. $PilB_{Gm}$ clusters more closely than $PilB_{Tt}$ with the PilB homologs from *Myxococcus xanthus*, *Pseudomonas aeruginosa*, and *Neisseria meningitidis*.

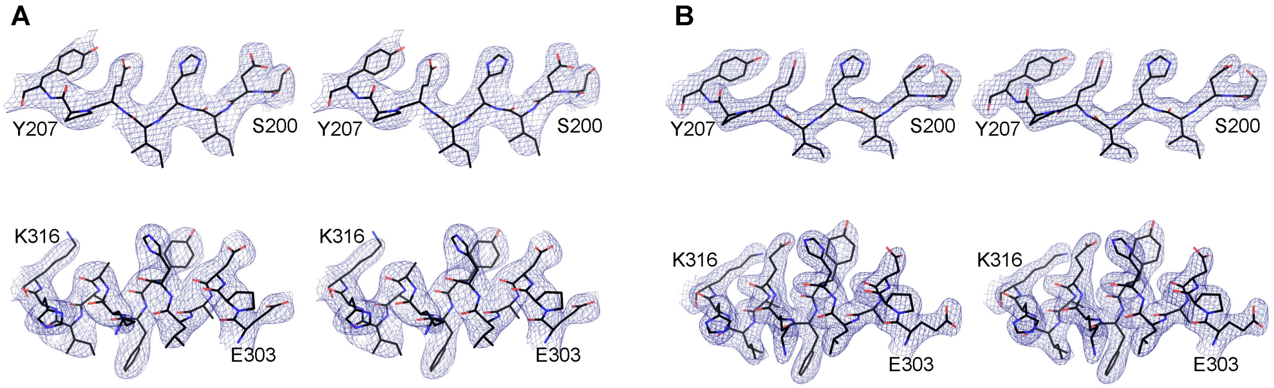

Supplementary Fig. 2. Stereo images of two portions of the electron density from PilB:ADP and PilB:AMP-PNP. 2Fo-Fc maps contoured at 1σ are shown as blue mesh. (A) Residues 200 to 207 and 303 to 316 in PilB:ADP. (B) Residues 200 to 206 and 303 to 316 in PilB:AMP-PNP.

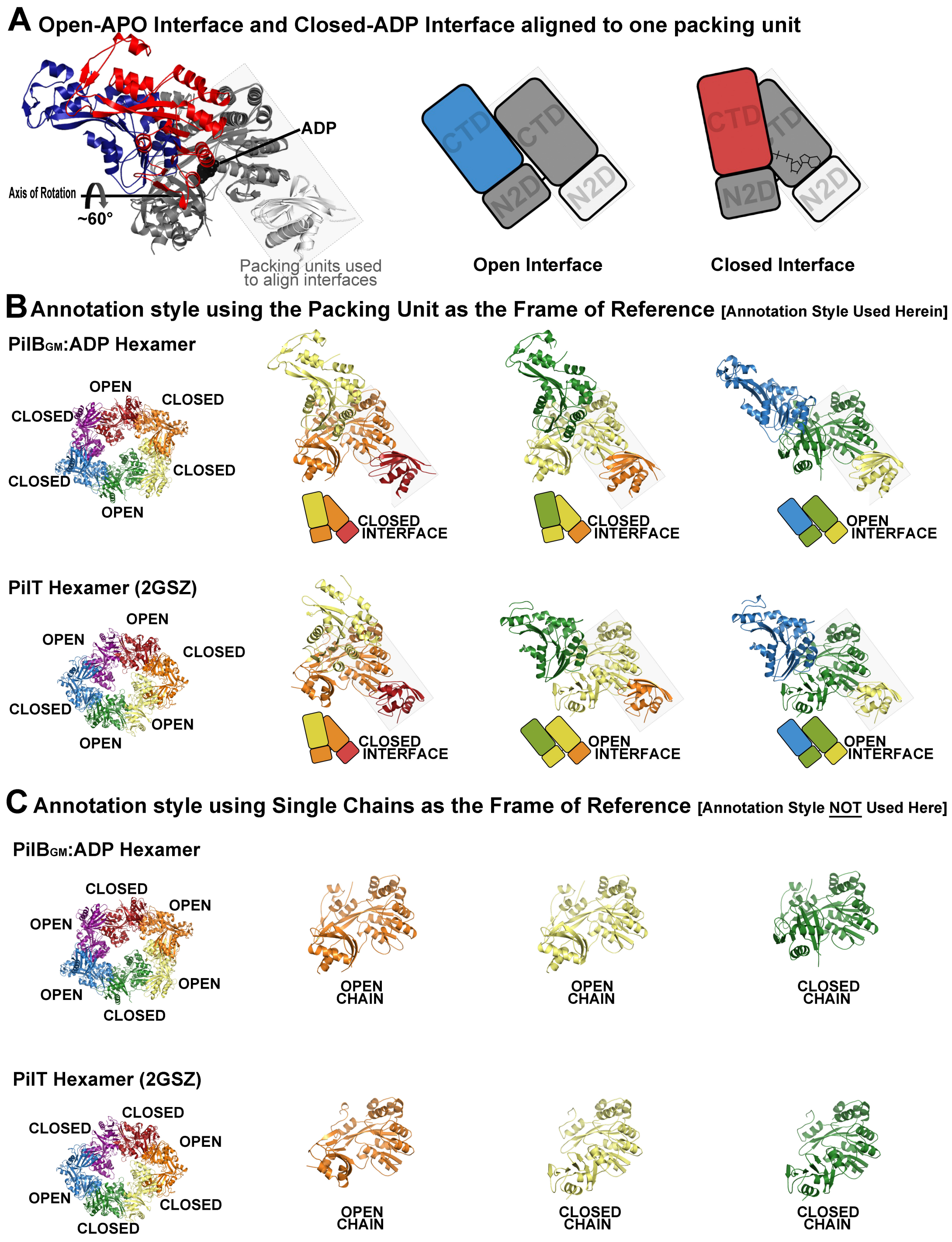

Supplementary Fig. 3. Defining the open and closed interfaces of PilB and PilT. (A) Alignment of the open-APO and closed-ADP packing interfaces from PilB:ADP. The axis of rotation identified by DynDom[26] is indicated. The light grey box illustrates the packing unit from each pair of packing units used to align the structures. ADP from the closed-ADP interface is shown as black spheres for reference. **(B)** On the left, the PilB and PilT hexamers are coloured as in Figure 2. The interfaces between representative pairs of packing units are shown on the right. The interfaces are shown in a similar format and orientation to panel A. The three interfaces shown are representative of the six interfaces in the C2 symmetric structures of PilB and PilT. **(C)** For comparison, three individual chains are shown that are representative of the six chains in the C2 symmetric structures of PilB and PilT. This style of annotation is not used here, but has been used elsewhere and is included for comparison purposes only[18,23].

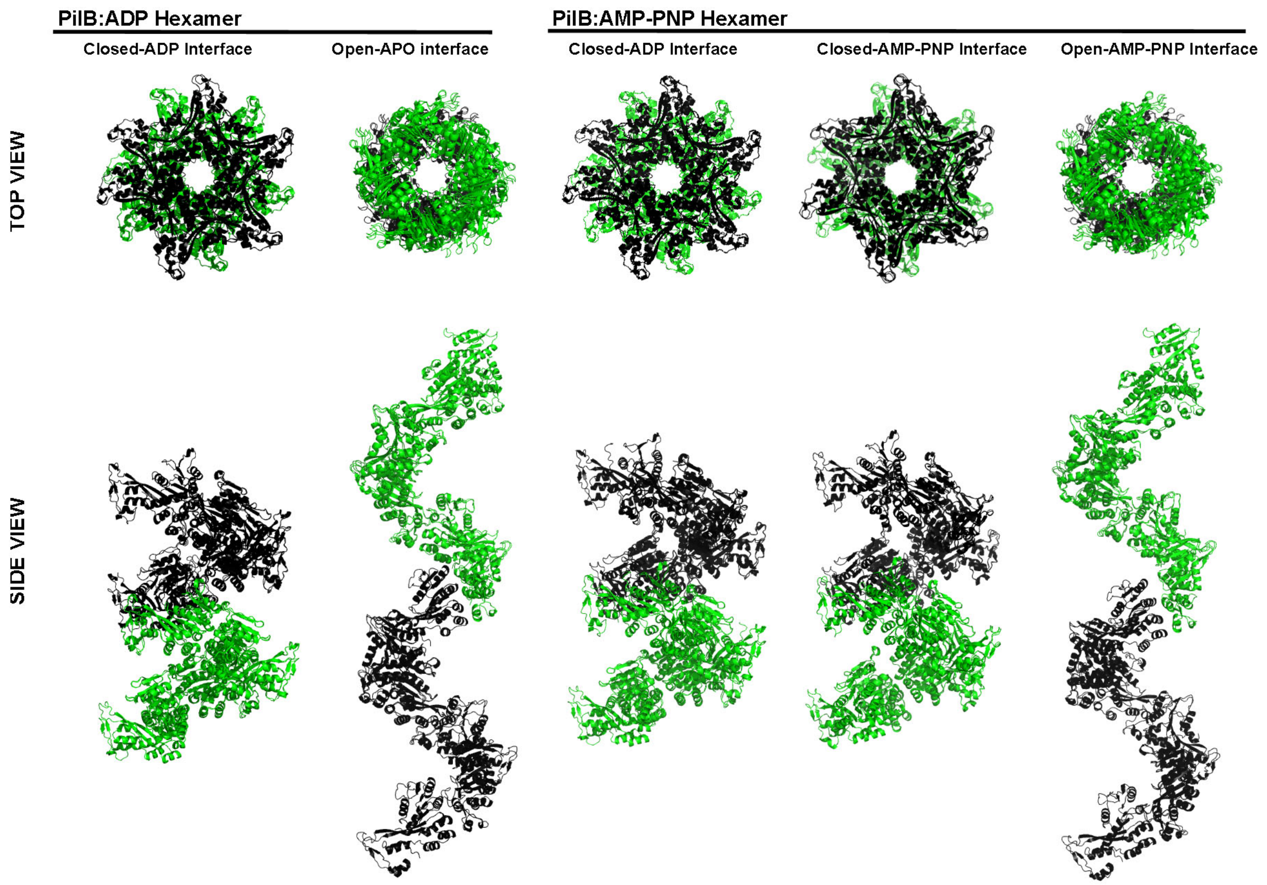

Supplementary Fig. 4. Helical properties of the packing unit interfaces in PilB. Twelve packing units were aligned to simulate a helix formed by the specified PilB interfaces. For simplicity, only the C2D of each packing unit is shown, the first six units are coloured black, while the last six are coloured green. The twist and rise of the closed-ADP interface from the PilB:ADP hexamer and the PilB:AMP-PNP hexamer was ~65° and -12 Å, respectively. The twist and rise of the open-APO interface from the PilB:ADP hexamer was ~76° and +24 Å, respectively. The twist and rise of the closed-AMP-PNP interface from the PilB:AMP-PNP hexamer was ~62° and -12 Å, respectively. The twist and rise of the open-AMP-PNP interface from the PilB:AMP-PNP hexamer was ~72° and +24 Å, respectively.

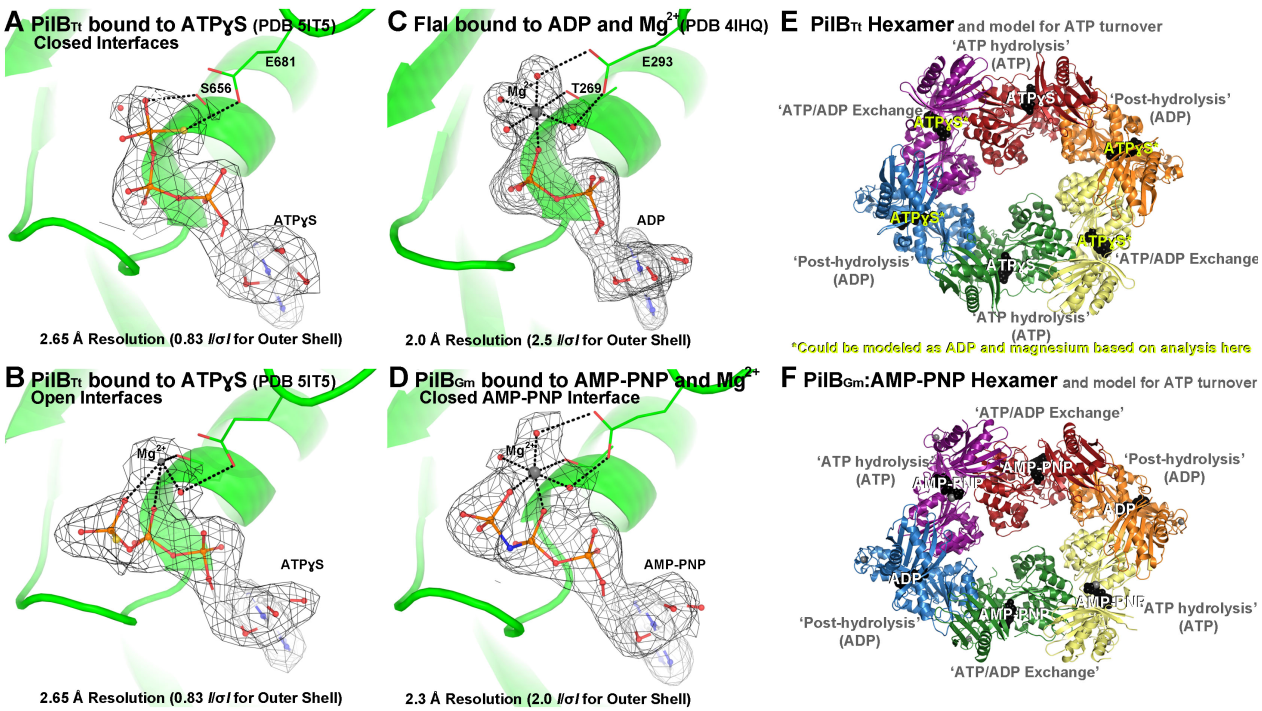

Supplementary Fig. 5. Comparison of the $PilB_{Gm}$ and $PilB_{Tt}$ models. Comparison of the ɣ-phosphate and magnesium electron density and model building between the **(A)** closed and (**B**) open packing interfaces of $PilB_{Tt}$, (**C**) a 2.0Å resolution PilT-like ATPase structure, FlaI (PDB 4IHQ and (**D**) closed-AMP-PNP interface of $PilB_{Gm}$:AMP-PNP. **(E)** Overview of $PilB_{Tt}$ hexamer, pattern of nucleotide binding, and model for ATP turnover generated by Mancl et al.[23] We have labelled in yellow the ATPγS molecules that – based on the higher resolution FlaI model – would more likely be ADP and magnesium. **(F)** Overview of $PilB_{Gm}$ hexamer, pattern of nucleotide binding, and model for ATP turnover as presented herein.

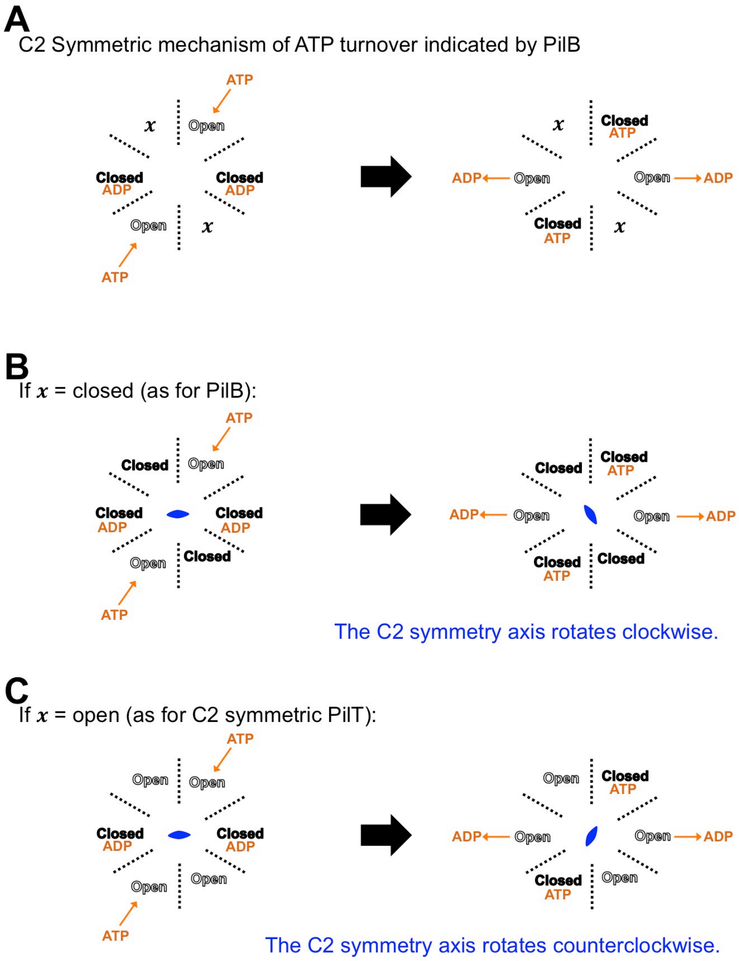

Supplementary Fig. 6. Cartoon describing how the enantimeric arrangement of open and closed interfaces in PilB and PilT would lead to opposite directions of elongated pore rotation. (A) the direction of ATP turnover indicated by the structures of PilB and the resulting changes in interface closure. (B), same as (A) if four interfaces were closed and two were open, as for PilB. (C), same as (A) if four interfaces were open and two were closed, as for PilT.
